# Goal Achievement Failure Drives Corticospinal Modulation in Promotion and Prevention Contexts

**DOI:** 10.3389/fnbeh.2018.00071

**Published:** 2018-04-24

**Authors:** Emanuele Lo Gerfo, Alberto Pisoni, Stefania Ottone, Ferruccio Ponzano, Luca Zarri, Alessandra Vergallito, Erica Varoli, Davide Fedeli, Leonor J. Romero Lauro

**Affiliations:** ^1^Department of Economics, Management and Statistics, University of Milano Bicocca, Milan, Italy; ^2^NeuroMI—Milan Center for Neuroscience, Milan, Italy; ^3^Center for Interdisciplinary Studies in Economics, Psychology and Social Sciences, University of Milano Bicocca, Milan, Italy; ^4^Department of Psychology, University of Milano Bicocca, Milan, Italy; ^5^Department of Political Science, Università del Piemonte Orientale, Vercelli, Italy; ^6^Department of Economics, University of Verona, Verona, Italy; ^7^Department of Medicine and Surgery, University of Milano Bicocca, Milan, Italy

**Keywords:** MEP, mirror neurons, neuroeconomics, regulatory focus theory, goal achievement failure

## Abstract

When making decisions, people are typically differently sensitive to gains and losses according to the motivational context in which the choice is performed. As hypothesized by Regulatory Focus Theory (RFT), indeed, goals are supposed to change in relation to the set of possible outcomes. In particular, in a promotion context, the goal is achieving the maximal gain, whereas in a prevention context it turns into avoiding the greatest loss. We explored the neurophysiological counterpart of this phenomenon, by applying Transcranial Magnetic Stimulation (TMS) and recording the motor evoked potentials (MEPs) in participants taking part in an economic game, in which they observed actions conveying different goal attainment levels, framed in different motivational contexts. More than the actual value of the economic exchange involved in the game, what affected motor cortex excitability was the goal attainment failure, corresponding to not achieving the maximal payoff in a promotion context and not avoiding the greatest snatch in a prevention context. Therefore, the results provide support for the key predictions of RFT, identifying a neural signature for the goal attainment failure.

## Introduction

In humans, Motor Facilitation (MF) is an extensively studied phenomenon consisting in an increment of the primary motor cortex (M1) excitability due to the observation of an action. The main and most direct measure of M1 excitability modulation is the amplitude of motor evoked potentials (MEPs) recorded from the muscle involved in the observed movement (Fadiga et al., 1995). The neural mechanism underlying MF is attributed to the human mirror neuron system (hMNS; Fadiga et al., 1995), defined as the neuronal network mainly composed by the premotor cortex, the inferior frontal gyrus and the inferior parietal lobule, which is known to be engaged while both executing and observing the same action (Rizzolatti and Craighero, 2004). Studies exploring the properties of MF showed that it is dependent on the kinematics of the observed action, being influenced by its spatial and temporal dynamics and by the perspective from which the action is viewed (Maeda et al., 2002; Gangitano et al., 2004; Mc Cabe et al., 2015). Interestingly, further studies highlighted how MF can be influenced also by the action’s *goal*, thus shedding light on the crucial role of the hMNS in recognizing action goals and agents’ intentions (Umiltà et al., 2001; Iacoboni, 2005; Cattaneo and Rizzolatti, 2009; Cavallo et al., 2012). A recent study (Pisoni et al., 2014) reported that MF can also be modulated by the *economic meaning* of the observed action. In particular, Pisoni et al. (2014) used two modified versions of the well-known dictator game (Kahneman et al., 1986; Camerer, 2003) in which a fictitious dictator decided whether sharing or not a gain or a loss with the experimental subjects which played as passive receivers. During the game, the dictator’s choices were communicated to the experimental subject through a video showing a right hand grasping one of two objects, each associated with opposite economic outcomes (Gain/no Gain or Loss/no Loss). Despite being the kinematics and the perspective of the observed action always the same, losses and gains elicited greater MEPs as compared to non-losses and non-gains. According to the authors, what mostly influenced the MEPs’ amplitude was a status quo modification, namely the outcomes leading to a variation of the subjects’ initial endowment, regardless of the economic impact of such change.

However, so far, whether MF could be sensitive to another factor playing a key role in socio-economic interactions, i.e., goal *achievement* vs. *failure* is still an open question. In particular, in economic exchanges, goal achievement assumes different meaning according to the motivational context in which the interaction occurs, i.e., aiming at avoiding the greater loss in prevention contexts and aiming at achieving the greater gain in promotion ones. Many daily activities—from attending a different school or moving to a new city to investing in the stock market or in a new entrepreneurial initiative—involve experiencing potential gains or losses relative to a given reference frame. Prior economic research supporting Prospect Theory (PT) indicates that, generally, subjects tend to treat losses and gains differently (Kahneman and Tversky, 1979; Thaler and Johnson, 1990; Tversky and Kahneman, 1991). However, when the choice is among options with equal outcomes, very different decisions can occur depending on the choice being framed in a loss or gain context (Tversky and Kahneman, 1985). In particular, abundant empirical evidence supports one of the key constructs developed within PT: individuals exhibit “loss aversion”, in the sense that losses are subjectively weighted more than equivalent gains (Tversky and Kahneman, 1991).

More recently, Regulatory Focus Theory (RFT; Higgins, 1997; Cesario et al., 2008) proposes a goal-attainment perspective, positing that individuals actively pursue some desired reference points in gain domains and move away from undesired end-states in loss domains (Idson et al., 2000; Sacchi and Stanca, 2014; for review Lanaj et al., 2012). According to RFT, thus, individuals seek “promotion”, aiming at achieving the maximum advantage, in the gain domain, while they seek “prevention”, aiming at avoiding maximum cost, in the loss domain (see e.g., Higgins, 1997, 1998; Idson et al., 2000; Zhao and Pechmann, 2007). Empirical evidence suggests that this goal-attainment perspective influences people’s choices and subjective emotions about a specific outcome (Idson et al., 2000).

Therefore, we conjectured that if M1 responded to the economic goals of the observed actions, and people differently reacted to goal promotion and prevention, we might expect a MEP modulation related to individual experiences of success or failure in pursuing an economic outcome. In particular, we expect MEPs to be modulated by *deviations from goal attainment*, i.e., when participants fail to achieve the maximum gain, when they are in a promotion motivational context, and when they obtain the maximum loss, when they are in a prevention context.

In the present study we tested this hypothesis by means of a paradigm in which the meaning of an observed action was manipulated by conveying either economic goal achievement or failure in different motivational contexts. Past research has shown that the regulatory focus system can be activated by specific tasks and stimuli (e.g., Higgins et al., 1994). In particular, a loss frame (loss and non-loss options) activates a prevention focus, whereas a gain frame (gain and non-gain options) activates a promotion focus (Shah et al., 1998), even without an active intervention of the experimental subject. Building on this, we used a modified version of the dictator game (Kahneman et al., 1986), that we termed the Share Game (SG), to generate both a prevention and a promotion context, by allowing the dictator to share a 50 tokens (equivalent to 1.25 €) gain or loss, respectively. Therefore, the promotion context occurred when the dictator was sharing a gain (gain condition), whereas the prevention context was induced by the sharing of a loss (loss condition). Moreover, when sharing both the gain and the loss of 50 tokens, the dictator could choose among three sharing options, corresponding to 10, 25 and 40 tokens. Among these options, the first and the latter are clearly the more extreme options, whereas the middle one could be considered moderate, for both gains and losses. Our experimental subjects (the receivers) were required to passively face the dictator’s choices. Based on RFT theory, we should expect a MEP modulation according to whether the dictator choice corresponded to the participants’ goal, which would be set on achieving the maximum gain in the promotion context (preferred option: gaining 40 tokens in losing) and avoiding the greatest loss in the prevention context (preferred option: 10 tokens). No clear expectation on how modulation would affect MEPs values was possible, due to the lack of clear-cut interpretation in the literature about motivation and emotion influence on MEPs modulation. We did not expect, instead, any modulation of MEPs recorded in mid-quantity trials. Predictions based on PT, instead, expected losses to produce a greater MEPs modulation, regardless of the quantities involved, thus including the mid-quantity options.

## Materials and Methods

### Participants

Twenty-four students (11 males, mean age = 23.29 years, SD = 1.87 years) recruited at the University of Milano-Bicocca took part in the experiment. Participants were right-handed, as assessed by the Edinburgh handedness inventory (EHI; Oldfield, 1971). They were naïve as to experimental procedure and aim of the study. All subjects were healthy and had no contraindication to transcranial magnetic stimulation (TMS; Wassermann, 1998). Written informed consent was obtained from all participants, who were paid for their participation. The experimental protocol was approved by the ethical committee of the University of Milano-Bicocca and was carried out in accordance with the ethical standards of the revised Helsinki Declaration (World Medical Association, 2013).

### TMS and EMG Recordings

TMS was applied using an Eximia TMS stimulator (Nexstim, Helsinki, Finland) using a focal bi-pulse, figure of eight 70-mm coil. The coil was positioned tangentially to the scalp over M1 hand knob, perpendicularly oriented to the central sulcus, which has been shown to be optimal for trans-synaptic activation of the cortico-spinal pathways (Brasil-Neto et al., 1992; Mills et al., 1992). Coil positioning and orientations were determined by means of an Neuronavigated Brain Stimulation system (NBS, Nexstim™, Helsinki, Finland) employing infrared-based frameless stereotaxy to map the position of the coil referenced to the participant’s head. Our study is one of few neurophysiological studies that monitored the coil position during each trial. The NBS provides an online visual information which warns with a visual cue if the coil position deviates more than 2 mm from the hotspot. Therefore, the NBS system strengthens our neurophysiological data eliminating any interference from coil position and orientation variations.

The coil was moved over the left M1 hand knob in order to identify the TMS hotspot, defined as the point where stimulation evoked the largest MEP from the contralateral first dorsal interosseus (FDI) muscle. Participants were seated in a comfortable chair and their right hand stayed in a relaxed position on their right thigh. TMS was delivered at 110% of the individual motor threshold (Loporto et al., 2013), assessed as the minimum intensity of the stimulator output required to induce MEPs of at least 50 μV of amplitude in the contralateral FDI muscle in at least five out of 10 trials (Rossini et al., 1994). MEPs were recorded from the FDI muscle of the right hand using 9-mm diameter Ag–AgCl surface cup electrodes. The active electrode was placed over the muscle belly and the reference electrode over the metacarpophalangeal joint of the index finger. Responses were amplified with eXimia EMG (Nexstim™, Helsinki, Finland) amplifier, filtered with a band pass of 10–500 Hz and digitized at a sampling rate of 3 kHz.

### Experimental Design and Procedure

The experimental procedure was divided in three phases.

#### Phase 1: Social Value Orientation measurement

In phase 1 the participants earned their initial endowment for the SG as a reward for their ability to play a brief attentional dummy task on a computer screen. This task required the discrimination of the orientation of a T letter in a circular array of L letters. All the participants were endowed with 500 tokens for their ability.

A relevant factor affecting people reactions to another player’s choice when playing an economic game is prosociality, which can be defined as the concern people have for others (Bieleke et al., 2016; for prosocial behavior in economic games, see also Camerer, 2003). In order to control for the impact of this dimension, after the attention task we measured a trait of prosociality in the participants using the SVO Slider Measure (Murphy et al., 2011)_._ SVO measure was obtained from the choices made during a series of dictator games in which each subject played the role of the dictator and decided how to allocate a certain amount of money between himself/herself and another person. The possible outcomes of their decisions were the following: maximizing the other’s payoff, which can be viewed as a behavior reflecting perfect altruism (i.e., the highest possible degree of altruism); maximizing one’s own payoff, which indicates individualism or selfishness; maximizing the difference between the own and the other one’s payoff, reflecting perfect competitiveness; minimizing the difference between payoffs or maximizing joint gains, indicating prosociality (for additional details see Murphy et al., 2011).

#### Phase 2: Share Game as Player A

In phase 2, in order to assess the precise desired economic goal of participants within the frame of the SG, participants played a computerized SG, implemented in two Regulatory Focus contexts, i.e., a gain and a loss condition, such to prompt either a promotion or prevention context respectively.

SG involved two types of players A and B: an active player (A, the dictator), in this phase our experimental subject; a passive player (B, five dummy subjects). In the gain condition, corresponding to a promotion context, player A decided how to share a 50 tokens reward with player B, choosing one out of three options (+40/+10, +25/+25 and +10/+40, where the first and the second number indicate player A and player B part of the sum respectively). In loss condition, corresponding to a prevention context, player A decided how to share a 50 tokens loss with player B by choosing one out of three options (−40/−10, −25/−25 and −10/−40). Each participant was informed that the computer was connected via internet to another experimental room with other players. Participants were also informed that each player B would also play as player A in phase 3. The SG in this phase lasted 20 trials, hence participants were playing four trials with each of the five players B. In each trial, player A did not know which player B s/he was playing with. At the beginning of each trial, the label “+50” or “−50” appeared on the screen, anticipating the incoming condition i.e., whether he/she was going to share a gain or a loss, respectively. A key on the keyboard was assigned to each possible option for player A (i.e., +40/+10, +25/+25 and +10/+40 in gain condition and −40/−10, −25/−25 and −10/−40 in loss condition). As soon as player A decided how to share the gain/loss with player B, s/he communicated her/his decision by pressing the corresponding key. At this point, a video clip showing the right hand of an actor grasping a metal cylinder (diameter 2 cm; height 1.5 cm) labeled with the chosen division scheme among all possible share options was displayed. Ten trials in the loss condition and ten trials in the gain condition were presented in a random order. All tokens earned during phase 1 constituted the subject’s endowment for phase 2. For each participant, we collected the percentage of sharing options chosen during the game.

#### Phase 3: Share Game as Player B

**Figure 1:**
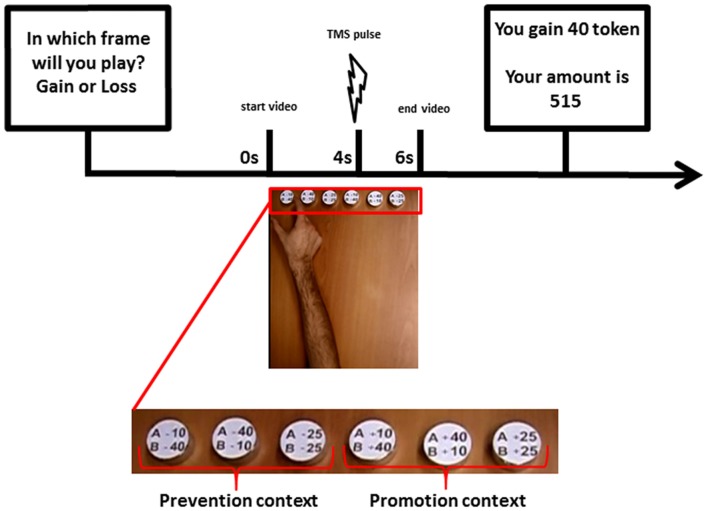
Timeline of an experimental trial in the Share Game (SG; Phase 2): At the beginning of each trial, the participants were asked to declare their expectation about the incoming condition of gain or loss. Then player A’s decisions were communicated through a video of an actor’s hand reaching and grasping a token representing one of the possible sharing options. In each trial, synchronized with the presented grasping action, a transcranial magnetic stimulation (TMS) pulse was delivered to the participant’s primary motor cortex (M1) and the elicited motor evoked potential (MEP) was recorded. A screen reporting the amount of tokens won or lost by player B up to that moment was then displayed at the end of each trial.

In phase 3, the main experimental phase, participants changed role and played as player B in the SG for 150 trials—30 trials with each of the other five dummy players of phase 2. At the beginning of each trial, participants were asked to declare their expectation about the incoming condition of gain or loss by pressing one of the two mouse keys with their left hand. This passage was introduced to record subjects’ expectations and to keep their attention focused on the task. Participants were informed that gain or loss conditions were generated in random order and that their prediction could not exert any effect. Exactly as in phase 2, player A’s decisions were communicated through a video of an actor’s hand reaching and grasping a token representing one of the possible sharing options. In each trial of phase 3, synchronized with the presented grasping action, a TMS pulse was delivered to the participant’s M1 and the elicited MEP was recorded. A screen reporting the amount of tokens won or lost by player B up to that moment was then displayed at the end of each trial (see Figure 1). In order to maintain a sustained attention during the task, in 30% of the trials subjects were asked to report player A’s choice. Subjects’ verbal answer was recorded by the experimenter.

Both gain and loss conditions and the sharing options chosen by the dummy player A were presented in random order and counterbalanced. More specifically, each share option (+10/+40, +25/+25, +40/+10, −10/−40, 25/−25, 40/−10) occurred for 25 trials for a total of 150 trials.

It is worth noting that, in this phase, playing as player B, participants could face three levels of goal attainment according to the corresponding sharing option chosen by the dummy player A: no goal (+40/+10), medium (+25/+25) and maximum (+10/+40) goal attainment, in the gain sharing condition, since the promotion perspective prompts the goal of achieving the maximal gain. In the loss condition, corresponding to the prevention context, player A decided how to share a 50 tokens loss with player B by choosing one out of three options (−40/−10, −25/−25 and −10/−40). Also in prevention context, player B could face three levels of goal attainment: no goal (−10/−40), medium (−25/−25) and maximum (−40/−10) goal attainment, since in this perspective the goal was to avoid the maximal loss. In both contexts, player B could not actively react to any decision taken by player A.

Participants were paid 0.025 € for each token they earned in the experimental phase. The average payoff was 19.6 €.

### Visual Stimuli

Stimuli consisted of a set of digital video clips presented on a 19-in LCD screen placed approximately 80 cm from the subject’ head. Videos showed, in egocentric view, the right hand of an actor, grasping one of six metal cylinders (diameter 2 cm; height 1.5 cm) horizontally placed on a table, at 57 cm from his hand. Egocentric view was chosen as it was the best perspective to elicit MF (Maeda et al., 2002). On each cylinder, one of the possible share options was impressed, resulting in three cylinders for the gain condition (40/10; 25/25; 10/40) and other three cylinders for the loss condition: −40/−10; −25/−25; −10/−40). The share option impressed on each cylinder indicated the dictator’s amount (player A) first, and the receiver’s amount (player B) second. In the video all the six cylinders were displayed, aligned in a row, with the three cylinders for each condition (gain and loss) on a side of the table respect to a midline centered in the video (see Figure 1). The side of the two contexts was counterbalanced across subjects, so that for half of participants the three cylinders representing the loss condition were placed in the right side and the cylinders representing the gain condition were placed on the left side. In this way, for each subject, a clear association between the promotion or prevention context and the side of the corresponding tokens was created. Indeed, as far as the video started, the direction of the movement of the actor’s hand toward the right or the left side of the screen immediately indicated whether the subject was going to share a gain or a loss, prompting the promotion or prevention context respectively. In addition, the order of the share options within both loss and gain conditions was balanced across subjects.

### Control Experiment

To exclude that modulations of MF were not attributable to unspecific effects of the experimental procedure, a control experiment was performed following the same procedures adopted for the main experiment, but recording MEPs also from the right Abductor Digit Minimi (ADM), a muscle not involved in the observed action. Ten participants, not recruited for the main experiment, took part in this control procedure (6 males, mean age = 23.9 years, SD = 1.6 years). In this session, the coil was positioned over the ADM hotspot following the previously described procedure. For ADM, the active electrode was placed over the muscle belly and the reference electrode over the metacarpophalangeal joint of the right pinky finger.

Additionally, in order to investigate if MEP modulation due to action observation represented an inhibition or a facilitation of the normal M1 excitability, in this supplementary experiment we collected a baseline measurement, of 30 trials, before the beginning of the experimental phase. During baseline recordings, participants observed the same video clips, with a hand grasping one out of six metal cylinders, but without the sharing options impressed on them.

### MEPs Preprocessing

MEPs data (phase 3 and control experiment) were processed off-line. Trials showing electromyographic activity prior to TMS pulse were removed from the analysis (Avenanti et al., 2006; Catmur et al., 2011). In each trial MEP amplitude was measured peak-to-peak (in mV). Trials in which MEPs exceeded ±2 SD of the subject’s mean were identified and removed. Based on this criterion, 12% of trials were removed in the main experiment, and 6.5% in the control experiment. The number of discarded trials did not differ among conditions (*p* = 0.947).

### Statistical Analyses

Data were analyzed in the statistical programming environment R (R Development Core Team, 2014).

To test how subjects made their choices in the SG in Phase 2, in which they played as player A, a Poisson regression was performed with number of choices per condition as DV and sharing options (6 levels: gain +10, +25 or +40 tokens and loss −10, −25 or −40 tokens) as IV.

To analyze the MEPs recorded during the SG in phase 3, in which participants were players B, linear mixed effects models were used as the main statistical procedure (Baayen et al., 2008). With this procedure, it is possible to account for inter-subject variability, which is prominent in MEP measurements, by adding a by-subject random slope (see Baayen et al., 2008). As our data involved a continuous dependent variable, logarithmic transformed MEP values were submitted to a series of linear mixed effects regression using LMER procedure in “lme4” R package (version 1.1-5, Bates et al., 2014). Log transformation was performed to increase the fit of our data to a normal distribution, without affecting parameter estimation. Fixed effects inclusion in the final model has been tested with a series of likelihood ratio tests, including each effect which significantly increased the model’s goodness of fit (Gelman and Hill, 2006). As fixed effects, we included the Share amount (factorial, 3 levels: 10, 25 and 40 tokens), the motivational Regulatory Focus context (factorial, 2 levels: promotion vs. prevention) and their interaction; as covariates we included Expectation (factorial, 2 levels: gain vs. loss), the responses given at the SG in phase 1 and the scoring at the SVO questionnaire. Concerning the random effect structure, a by-subjects and a by-items random intercept were included. Moreover, the inclusion of a by-subjects and by trial random slope for Share amount and motivational Regulatory Focus context and their interaction were tested (Matuschek et al., 2015; Bates et al., 2015 ). We report the LRT procedures outcomes for model selection and parameters of the final best fitting model models, together with significance level based on Satterthwaite’s degrees of freedom approximation in lmerTest R package (version 2.0-6, Kuznetsova et al., 2015; Tables 1, 2). Significant interactions were explored using the R package “phia” (Martinez, 2015) applying FDR correction for multiple comparisons.

**Table 1:** Model selection: LRT results.

	*DF*	χ^2^	*p*
**Fixed effects**			
**Effect**
Dictator	1	0.43	0.51
Expectation	1	1.61	0.2
SVO	1	0.72	0.4
RF Context	1	0.29	0.59
Share amount	2	0.029	0.98
RF Context*Share amount	2	14.76	0.0006*
**Random effects**			
**Trial**			
Share amount*RF Context	11	1.81	0.99
RF Context	4	0.72	0.95
Share amount	7	7	0.42
ID			
Share amount*RF Context	NA	NA	NA
RF Context	2	5.72	0.057*
Share amount	5	3.6	0.6

*Asterisks identify parameters which significantly increased the goodness of fit of the model*.

**Table 2:** Final model parameters.

Random effects	SD	corr		*n*	
Trial	0.12			180	
ID	0.498			24	
RF Context|ID	0.01	−0.41		
**Fix effects**	**Est**	**SE**	**DF**	***t***	***p***
Intercept	6.5	0.1	25.7	62.9	<0.001
Share amount 10:25	−0.015	0.4	3028.7	−0.37	0.71
Share amount 10:40	0.08	0.4	3042.9	1.11	0.03*
Share amount 25:40	0.1	0.04	3030	2.47	0.013*
RF Context Promotion:Prevention	0.04	0.45	96.8	0.92	0.36
Share amount 10:25* RF Context Promotion:Prevention	0.023	0.057	3038.7	0.4	0.69
Share amount 10:40* RF Context Promotion:Prevention	−0.18	0.0057	3036.3	−3.15	0.002*
Share amount 10:40* RF Context Promotion:Prevention	−0.2	0.0057	3020.3	−3.54	<0.001*

LN ~ Share amount * RF ContextV_P + (1 + RF ContextV_P | ID) + (1 | Trial). Asterisks identify significant parameters.

## Results

### Phase 1

SVO Slider Measure showed that 80% of our sample was formed by subjects with a preference for prosociality.

### Phase 2

**Figure 2:**
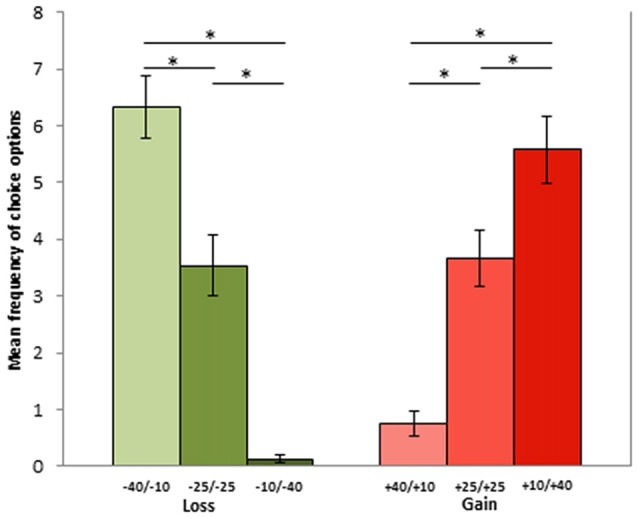
Mean frequencies for each choice option during phase 1. Options represent −40/−10, −25/−25 and −10/−40 in the loss, and +40/+10, +25/+25 and +10/+40 in the gain context. The first and the second number indicate player A and player B part of the sum, respectively). Asterisks represent significant differencies with *p* < 0.05.

Concerning phase 2 results, the Poisson regression on the SG highlighted a significant effect of condition ($\chi_{\left(5\right)}^{2}$ = 221; *p* < 0.001). In particular, losing 10 tokens was more often adopted (6.33 times) as compared to losing 25 (3.5 times; *p* < 0.001) or 40 tokens (0.12 times; *p* < 0.001), as losing 25 was more often chosen than losing 40 (*p* < 0.001). Similarly, gaining 40 tokens was more frequently chosen (5.58 times) than gaining 25 (3.66 times; *p* = 0.033) or 10 tokens (0.75 times; *p* < 0.001), as gaining 25 tokens was more often opted for than gaining 10 tokens (*p* < 0.001). There was no difference in gain and loss conditions when the economic outcome of the exchange was the same (gain 10 vs. lose 40: *p* = 0.06; gain 40 vs. lose 10: *p* = 1; gain 25 vs. lose 25: *p* = 1). Conversely, gaining 40 was much more frequently chosen than losing 40 tokens (*p* < 0.001), and losing 10 was more frequently chosen than gaining 10 tokens (*p* < 0.001; see Figure 2).

### Phase 3. Main Experiment

Concerning the neurophysiological results of the SG in phase 3, the final model on MEP amplitude included the main effects of Share amount, motivational RF contexts and their interaction as fixed effects, while the random effects structure included the random intercept for Trial and Subject, as well as a by Subject random slope for motivational RF context.

**Figure 3:**
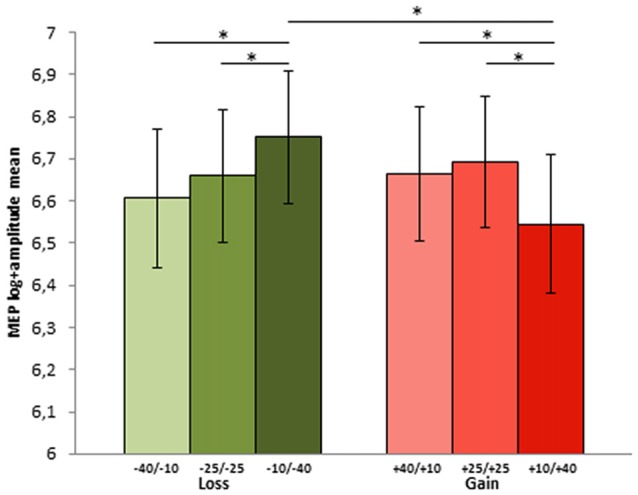
MEP log-amplitude mean for each choice options in the two contexts during phase 2 (−40/−10, −25/−25 and −10/−40 in the loss and +40/+10, +25/+25 and +10/+40 in the gain context. The first and the second number indicate player A and player B part of the sum, respectively). Error bars represent ± MSE. Asterisks indicate significant differences at *p* < 0.05.

The parameters of the final model highlighted a significant effect of share amount, with difference between MEPs elicited in the highest share amount condition (40 tokens, 1.73 mV) as compared to both the 10 (*b* = 0.08, *t*_(3042.9)_ = 2.11; *p* = 0.03) and 25 tokens (*b* = 0.1, *t*_(3030)_ = 2.47; *p* = 0.013) conditions (1.61 mV and 1.51 mV, respectively). Interestingly, the final model included also the interaction between the share amount and the motivational RF contexts: in both RF contexts the 40 tokens amount was significantly different from both the 25 (*b* = −0.2, *t*_(3020.3)_ = −3.54; *p* = < 0.001) and 10 (*b* = −0.18, *t*_(3036.3)_ = −3.15; *p* = 0.002). Within the prevention motivational RF context, prompted by loss condition, the 40 tokens loss share (sharing option −10/−40), which represented the goal attainment failure for this context, elicited greater MEPs both compared to the 25 (sharing option −25/−25; *p* = 0.038) and 10 (sharing option −40/−10; *p* = 0.05) tokens shares. Conversely, in the promotion motivational RF contexts, in the gain condition, 40 tokens shares (option +10/+40), which represented the maximal goal attainment, elicited smaller MEPs compared to the 25 (option +25/+25; *p* = 0.038) and 10 (option +40/+10) *p* = 0.038) tokens shares (see Figure 3), which instead deviated from the desired outcome.

Comparing the two motivational RF contexts for each amount, while for the 40 token the prevention context elicited greater MEPs (1.15 mV) compared to the promotion context (0.99 mV; *p* = 0.007), no difference between the two contexts was found for the 25 (1.03 mV vs. 1.07 mV; *p* = 0.23) and the 10 (1.06 mV and 1.065 mV; *p* = 0.36) tokens shares.

### Control Experiment

Concerning supplementary experiment neurophysiological results, the model on MEP amplitude of ADM did not include neither the main effects of Share amount ($\chi_{\left(2\right)}^{2}$ = 0.072, *p* = 0.96;) and Regulatory Focus context ($\chi_{\left(1\right)}^{2}$ = 0.146, *p* = 0.7), nor their interaction ($\chi_{\left(2\right)}^{2}$ = 0.588, *p* = 0.74).

Finally, we performed a model on MEP’s amplitude of ADM and FDI including condition (2 levels: baseline-MEP recorded during the view of videos without share options impressed on the six metal cylinders and experimental MEP recorded during experimental phase) as fixed effects, while the random effects structure included the random intercept for Trial and Subject. Model highlighted a significant difference between baseline and experimental conditions for both ADM: $\chi_{\left(1\right)}^{2}$ = 207.73, *p* < 0.001 (mean 0.476 mV in baseline condition, vs. 0.853 in experimental condition) and FDI $\chi_{\left(1\right)}^{2}$ = 30.106, *p* < 0.001 (mean 1.234 mV in baseline condition, vs. 1.713 in experimental condition).

## Discussion

In the present study, we investigated the effects of economic goal attainment and failure, which are central notions within the so called RFT, on motor corticospinal excitability during economic exchanges, by considering both promotion and prevention contexts. The context of promotion was created asking participants to face the choice of a dictator in the condition of sharing a gain, whereas the prevention context was generated by analyzing the share of a loss. We started by assessing the normal behavior of our participants in sharing an economic gain or loss by assigning them to the role of dictators. This allowed us to evaluate which goal the participant wanted to actively pursue in both promotion and prevention contexts. Consistently with our predictions based on RFT, subjects tended to seek the minimum loss (−10) in the prevention context and to achieve the maximum gain (+40) in the promotion frame (phase 2 results, see Figure 2). Interestingly, there was no difference in the choice rate when the economic value of goal achievement expected in the two contexts was comparable (i.e., +10 vs. −40, +25 vs. −25 and +40 vs. −10 tokens).

When participants played as passive receivers of the SG (player B, phase 3), the maximum MEP amplitude was associated with sharing thei less desired options, as indicated by the choices made during phase 2 and in line with the predictions of RFT. More specifically, in the prevention context, i.e., when subjects aimed at reducing the loss, the highest MEPs were recorded when participants were given the −40 token share (sharing option −10/−40), i.e., the most distant option from achieving their goal of minimizing the loss. In the promotion context, the greatest MEP amplitude was detected in trials in which the maximum gain (40 tokens, corresponding to option +10/+40) was not obtained by the participant. Conversely, achieving a goal in both promotion and prevention contexts (i.e., when the subjects gained 40 or lost 10 tokens) ended in lower MEP amplitude. To the best of our knowledge, this is the first evidence that corticospinal excitability is modulated by goal attainment during an economic game.

We therefore propose our neuroeconomic study as supporting the key predictions of RFT, which highlights the role of motivational intensity on the subjective value of a gain or a loss. More specifically, our data offer a neurophysiologic signature of RFT behavioral predictions, suggesting a role of the hMNS in modulating, coherently with RFT predictions, cortico-spinal excitability. Concerning the neural underpinnings of RFT, previous functional imaging studies revealed that promotion goal attainment exhibited activation in frontal regions as the medial prefrontal cortex and the anterior cingulate cortex (ACC), whereas prevention goal attainment was associated with activations in more caudal regions as the bilateral posterior cingulated cortex and the precuneus (Johnson et al., 2006; Sharon et al., 2007). Other studies indicated an involvement of amygdala, ACC and extrastriate cortex during verbal judgment in promotion and prevention contexts (Cunningham et al., 2005). Some of these regions share direct and indirect connections with segments of the putative hMNS, in particular with the premotor cortex (Leung et al., 2015). Furthermore, this complex network supports the idea that, being RFT a motivational theory, both cognitive evaluation and emotions are involved in the reactions and expectations linked to its different outcomes.

Accordingly, previous studies highlighted how motivation and emotion linked to monetary action observation may affect M1 neurophysiology. In particular, the presence of a monetary reward was found to modulate short intra-cortical inhibition (Thabit et al., 2011) and MEPs amplitude (Kapogiannis et al., 2008; Suzuki et al., 2014). Next, while the majority of studies focused on M1 modulation of positive outcomes, Vicario et al. (2015) investigated the effects on cortical excitability of both gains and negative economic outcomes. These authors found that monetary losses were associated with greater MEPs values when actively pursued by the experimental subjects and, critically, that outcome-related negative emotions increased with cortico-spinal output.

It could be argued that the modulation of the motor cortex activity was due to a negative emotional reaction to maximum losses and to non-maximum gains *per se*. RFT, indeed, being a motivational theory and thus including the effects of emotions on cognitive states, predicts that promotion success (gain) elicits cheerfulness-related emotions, while promotion failure (non-gain) produces negative emotions, such as low-intensity sadness. On the other hand, prevention success (non-loss) generates quiescence-related emotions, and failure in prevention focus (loss) induces agitation-related emotions (Idson et al., 2004). Earlier studies evaluated the impact of emotions on corticospinal excitability (Oliveri et al., 2003; Hajcak et al., 2007; Coombes et al., 2009; Coelho et al., 2010; Komeilipoor et al., 2013; Borgomaneri et al., 2015a,b). However, there is no clear-cut interpretation so far of how emotional stimuli may activate or suppress motor cortex excitability. Whereas some studies report an increase in MEPs while presenting unpleasant (Oliveri et al., 2003; Coombes et al., 2009; Coelho et al., 2010; Nogueira-Campos et al., 2016), or both pleasant and unpleasant emotional stimuli (Hajcak et al., 2007; Komeilipoor et al., 2013), others found a decrease in MEPs while subjects attended unpleasant emotional stimuli (Borgomaneri et al., 2015b) or no difference between unpleasant and neutral trials (Borgomaneri et al., 2012). Moreover, the majority of studies investigated the impact of emotions aroused by external stimuli (such as pictures or sounds) while only a few tested a possible role of internally generated emotional states (Vicario et al., 2015). In this view, the vastly reported increment in motor cortex excitability assessed with the concurrent presentation of emotional stimuli (whether they are pleasant or unpleasant) can be accounted for by current theories which posit preparation for action as a fundamental characteristic of the emotional state (Arnold, 1959; Frijda, 1988; Lang et al., 1990). In our study, however, only the negative emotional reaction, triggered by the failure in attaining personal economic goals, elicited an increase of M1 excitability. Interestingly, our study showed how contextual framing of motivation differently affects M1 excitability, increasing thus the degree of complexity underlying the relationship between emotions, economic exchanges and their neural signatures.

In addition, our results significantly expand the role of the hMNS in understanding the goal of an action in social interactions. The effect of action’s goal on MF has been already explored in previous studies, but the meaning of “goal” significantly differs across them. In turn, the end-goal of the observed action has been operationalized as grasping an object inserted in different contexts, or objects which could be visible or hidden, reachable or not reachable by the subject (Iacoboni, 2005; Gazzola et al., 2007; Umiltà et al., 2008; Urgesi et al., 2010; Cavallo et al., 2013). In other studies, instead, the goal of the observed action could entail a social meaning, such as reaching a ball to pass it to someone else (Bucchioni et al., 2013). In a previous work, we considered as “action meaning” its economic consequences in deviating from a status quo in terms of gain vs. non-gains and losses vs. non-losses (Pisoni et al., 2014). Conversely, in the present study we took into consideration not only the real end-goal of the observed action, but also its implicit goal, or better, its deviation from the desired maximal outcome, which is modulated by the promotion vs. prevention context in which the action is performed. RFT indeed predicts that the regulatory focus can be induced even in momentary situations: generally, gain and non-gain situations can induce a promotion focus, while loss and non-loss situations can induce a prevention focus (Idson et al., 2004). Promotion focus induces representation of the goal as the achievement of the best possible outcome, while prevention focus defines the goal as the avoidance of the worst option. In our study, the failure in achieving these goals generated an increase of corticospinal excitability in the experimental subjects.

Our neurophysiological data fit nicely with the prediction of RFT, while we consider them as only partially in line with the key tenet of PT, i.e., the notion of loss aversion (Tversky and Kahneman, 1981). Tversky and Kahneman (1981) proposed that the subjective experience of pain from a loss is greater than the experience of pleasure from a gain of the same entity. Whereas the difference between the MEP amplitude during the maximum loss (−40 tokens) and the maximum gain (+40 tokens) trials was significant, this was not true for intermediate levels of gains, where MEP values were comparable to intermediate levels of losses (25/25 vs. −25/−25), and for small gains, which did not differ from small losses (40/10 vs. −40/−10).

Several studies demonstrated the involvement of the arousal dimension in neuroeconomic studies, indicating that the arousal for a loss of a certain magnitude is greater than the arousal for a gain of the same magnitude (Tversky and Kahneman, 1985; Wu and Zhou, 2009). According to this hypothesis, we would have expected greater MEP amplitude in prevention context for all the levels of share amount, but this is true only for maximum amount. On the contrary, a previous study (Samanez-Larkin et al., 2007) underlined that the expectation of a great gain or a great loss raised the same level of arousal. On the basis of this last evidence we would have expected the same modulation of corticospinal excitability (CSE) for the maximum share amount, while our data shows a different pattern of modulation of CSE between promotion and prevention contexts. For this reason our results do not support a clear involvement of the arousal dimension.

Taken together, thus, the present results appear more in line with the goal-attainment perspective advanced by RFT. Finally, it has to be noted that our results are not directly linked to the size of the stake in each trial. If simply losing more money induced greater MEPs, we should have found differences in amplitudes between losing 10 and losing 25 tokens as well as between gaining 10 (and thus losing 40) and gaining 25 (thus losing 25) tokens. MEPs values, instead, were comparable in these conditions.

In conclusion, the present findings suggest that the hMNS is sensitive not only to the goal of the observed action, but also to one’s current goal pursuits (Huang et al., 2013) as framed by the motivational context posited by RFT. Our results provide support to the idea, advanced by RFT, that economic agents’ decision-making processes, far from being driven by context-independent goals, crucially depend on frame-specific, contingent, ends, i.e., trying to achieve a desired outcome vs. attempting to avoid an undesired outcome (Florack et al., 2013).

## Author Contributions

ELG designed research. ELG, AV, EV and DF performed research. AP conducted the EMG analysis, FP conducted SVO analysis. ELG, SO, AP and LZ prepared the draft and ELG, SO, LZ, FP, AP and LJRL jointly produced the final draft.

## Conflict of Interest Statement

The authors declare that the research was conducted in the absence of any commercial or financial relationships that could be construed as a potential conflict of interest.
